# The 2022 Vaccines Against *Shigella* and Enterotoxigenic *Escherichia coli* (VASE) Conference: Summary of abstract-based presentations

**DOI:** 10.1016/j.vaccine.2023.11.031

**Published:** 2024-03-07

**Authors:** Soumalya Banerjee, Eileen M. Barry, Shahida Baqar, A. Louis Bourgeois, Joseph J. Campo, Robert K.M. Choy, Subhra Chakraborty, Allison Clifford, Carolyn Deal, Marcus Estrada, James Fleckenstein, Mateusz Hasso-Agopsowicz, William Hausdorff, Ibrahim Khalil, Nicole Maier, Cynthia Mubanga, James A. Platts-Mills, Chad Porter, Firadausi Qadri, Michelo Simuyandi, Richard Walker, Jessica A. White

**Affiliations:** a ICMR – National Institute of Cholera and Enteric Diseases, India; b University of Maryland School of Medicine, United States; c US National Institutes of Health, United States; d PATH, United States; e Antigen Discovery, Inc, United States; f Johns Hopkins University, United States; g Washington University School of Medicine, United States; h World Health Organization, Switzerland; i University of Washington, United States; j Centre for Infectious Disease Research in Zambia, Zambia; k Division of Infectious Diseases and International Health, University of Virginia, United States; l Naval Medical Research Command, United States; m ICDDR,B, Bangladesh; n Faculty of Medicine, Université Libre de Bruxelles, Belgium

**Keywords:** Enteric diseases, Vaccine development, *Shigella*, Enterotoxigenic *Escherichia coli*, *Campylobacter*, Invasive non-typhoidal *Salmonella*

## Abstract

The global nonprofit organization PATH hosted the third Vaccines Against *Shigella* and Enterotoxigenic *Escherichia coli* (VASE) Conference in Washington, DC, on November 29 to December 1, 2022. With a combination of plenary sessions and posters, keynote presentations, and breakout workshops, the 2022 VASE Conference featured key updates on research related to the development of vaccines against neglected diarrheal pathogens including *Shigella*, enterotoxigenic *Escherichia coli* (ETEC), *Campylobacter*, and *Salmonella*. The presentations and discussions highlighted the significant impact of these diarrheal pathogens, particularly on the health of infants and young children in low- and middle-income countries, reflecting the urgent need for the development and licensure of new enteric vaccines. Oral and poster presentations at the VASE Conference explored a range of topics, including: the global burden and clinical presentation of disease, epidemiology, and the impact of interventions; the assessment of the value of vaccines against enteric pathogens; preclinical evaluations of vaccine candidates and models of enteric diseases; vaccine candidates in clinical trials and human challenge models; host parameters and genomics that predict responses to infection and disease; the application of new omics technologies for characterization of emerging pathogens and host responses; novel adjuvants, vaccine delivery platforms, and immunization strategies; and strategies for combination/co-administered vaccines. The conference agenda also featured ten breakout workshop sessions on topics of importance to the enteric vaccine field, which are summarized separately. This article reviews key points and highlighted research presented in each of the plenary conference sessions and poster presentations at the 2022 VASE Conference.

## Highlights

• The third Vaccines Against *Shigella* and ETEC (VASE) Conference was in Nov. 2022.
• Enteric diseases are a substantial public health burden in low-resource settings.
• VASE had an expanded scope that included other neglected diarrheal pathogens.
• Applying innovative tools can improve vaccine efficacy, delivery, and uptake.
• Vaccines against a broader scope of agents will help address diarrhea and AMR.

## 1 Introduction

From November 29 to December 1, 2022, the global nonprofit organization PATH hosted the third international Vaccines Against *Shigella* and Enterotoxigenic *Escherichia coli* (VASE) Conference in Washington, DC. With 270 attendees from more than 29 countries, it was the largest and most geographically diverse of the three VASE Conferences to date. *Shigella* and enterotoxigenic *Escherichia coli* (ETEC) were the primary focus for previous VASE Conferences, but PATH expanded the scope of VASE in 2022 to include research on other neglected enteric diseases among infants and children in low-resource settings, such as *Campylobacter*, non-typhoidal *Salmonella*, typhoid, cholera, and other diarrheagenic *E. coli*.

New findings covering a range of enteric vaccine topics were reported in 24 oral and 87 poster presentations based on submitted abstracts. In addition, the conference featured two keynote addresses. Dr. Celine Gounder of the Kaiser Family Foundation and Kaiser Health News presented on her experiences during the COVID-19 pandemic and lessons from the rapid development and roll-out of vaccines, which may be applicable to enteric vaccines. Dr. Kathleen Neuzil of the University of Maryland School of Medicine drew from her current work on typhoid conjugate vaccine introduction in low- and middle-income countries (LMICs) to highlight issues and opportunities relevant for developers of vaccines for other neglected enteric pathogens. There were also ten breakout workshop sessions (summarized separately in this issue of *Vaccine*), which directed smaller groups of conference participants to engage in deeper discussions on subjects of interest to the enteric vaccine field.

The following provides a brief summary of the research presented in each of the plenary sessions and the poster presentations at the 2022 VASE Conference in order to share the meeting content with the broader enteric vaccine field. The final conference agenda and abstracts booklet are available on the PATH website (www.path.org/resources/2022-vase-conference-abstracts-booklet/), and each presentation mentioned in this article is referenced using its assigned identifier code.

## 2 Global burden and clinical presentation of disease, epidemiology, and the impact of interventions

*Shigella* spp. continue to be a major bacterial enteric disease threat. Data from population-based surveillance in western Kenya from 2010 to 2015 demonstrated a 24% *Shigella* culture-positive rate among clinic patients with diarrhea, with an overall adjusted incidence of 7.9 episodes per 1,000 person-years and peak incidence of 15.3 episodes in children 12–23 months of age. Susceptibility to the antibiotics ceftriaxone and ciprofloxacin remained high (>98%) (GBD26). A deeper analysis of the Kenyan data further detailed the prevalence, serotype distribution, risk factors for illness, and antimicrobial susceptibility of *Shigella* (GBD16). *Shigella flexneri* remained the dominant species, with prevalence peaking in the second year of life. Similarly, data from Bangladesh from 2001 to 2020 showed that *Shigella* remained a common, if slightly declining, cause of diarrhea in urban and rural settings, with *S. flexneri* predominant. In both settings, ciprofloxacin resistance became common (>70%) and multidrug resistance significantly increased (>10%) by 2020 (GBD02). Another study that examined *Shigella* and a different class of antibiotics estimated that a 60% efficacious *Shigella* vaccine used in children could reduce fluoroquinolone and macrolide (F/M)-treated all-cause and *Shigella*-attributed diarrhea episodes by 9% and 46%, respectively, and F/M bystander exposure by 44% (AVV04).

Modeling studies added insight on the global distribution of shigellosis and the potential impact of a vaccine. A broad range of hydrometerological, environmental, household, and subject-level variables were used to perform spatiotemporal mapping of *Shigella* burden on a global scale. Based on these studies, the predicted prevalence of *Shigella* in child stool samples exceeded 25% in tropical sub-Saharan Africa, South and Southeast Asia, and limited regions of Central America and northern South America (GBD05). A study evaluating the potential impact and cost-effectiveness of *Shigella* vaccines in LMICs demonstrated a substantial impact, especially in Africa and in countries eligible for support from Gavi, the Vaccine Alliance. The inclusion of *Shigella*-attributable stunting further improved cost-effectiveness and extended the potential benefits to additional geographic regions (GBD28).

Clinical presentation and diagnosis of *Shigella* were addressed in Zambia (GBD09). While the classic shigellosis presentation is blood in stool, other clinical presentations are associated with *Shigella* infections as well, with 58% of the cases with watery diarrhea testing positive for *Shigella* and 68% presenting with mucus. Results from another study (GBD31) corroborated these results, finding that dysentery (bloody diarrhea) had a sensitivity of 8.5% in identifying *Shigella* in children by stool polymerase chain reaction (PCR). This high burden of shigellosis in Zambia also provided an opportunity for a new study to investigate its association with plasma biomarkers of environmental enteric dysfunction (EED), stunting, and the risk of subsequent *Shigella*-associated diarrhea in children (GBD12). These observations from Zambia are consistent with findings from a study in Kenya, Mali, and The Gambia, where *Shigella* was associated with a higher proportion of watery diarrhea compared to cases presenting with dysentery (GBD01).

Across pathogens, bacterial culture remains a critical tool for tracking antimicrobial susceptibility, performing serotyping, and potentiating genomic analyses. A culture optimization study identified buffered glycerol saline as a better transport media for *Shigella* than Cary Blair and highlighted the importance of rapidly transporting (<24 h) samples to the laboratory and maintaining the cold chain (4 °C) in improving recovery (GBD25). Better *Shigella* isolation also facilitates genomic analyses. A recent analysis of isolates from South Africa from 2011 to 2015 described *S. sonnei* and *S. flexneri* 2a as the dominant serotypes, but diverse sub-lineages with distinct patterns of antimicrobial resistance (AMR) and geographic distributions were also found to be circulating (GBD33). In related work from South Africa (GBD11), pathogen yields from hospital-based surveillance studies were significantly higher when molecular methods were used than when routine culture-based diagnostics were applied. The most significant difference in detection rates were noted for *Shigella* spp. (22.7% vs. 0.8%; p < 0.001), *Cryptosporidium* spp. (13.2% vs. 3.0%; p < 0.001), and *Campylobacter* spp. (11.8% vs. 0.2%; p < 0.001) when molecular methods and culture were compared, respectively. Separately, geospatial mapping of *Shigella* serotypes and AMR profiles was carried out using stool samples from Nigerian school children (PRE14). The data suggest continuous community spread of AMR *Shigella* strains, and the identification of focal outbreaks informs targeted interventions. Collectively, these data further highlight the value of more sensitive detection methods in assessing enteric disease burden. In comparative etiology studies from Brazil, the antigenic and virulence features of circulating strains of *Shigella* were assessed as they are important drivers of both vaccine development and uptake (GBD23). This work revealed that *S. sonnei* is the most prevalent species with *S. flexneri* still common, especially in association with shigellosis outbreaks. Moreover, the increasing prevalence of AMR among *Shigella* spp. makes effective treatment even more difficult.

ETEC was a significant pathogen in the first two years of life among children in Lima, Peru, causing one or more diarrhea episodes in 70% of children (GBD03). ETEC-attributable diarrhea (majority ST-ETEC) was high in neonates (10%) and peaked (17%) at 21–24 months of age. The most commonly occurring colonization factors (CFs) were CFA/I, CS12, CS21, CS3, and CS6. ETEC diarrhea was associated with stunting and underweight at the end of two years of follow-up. Similarly, ETEC diarrhea cases showed significantly lower mean weight-age Z-scores in children 6 months to < 3 years old in Cap-Haitien, Haiti (GBD13).

ETEC also contributed to a significant proportion of severe diarrhea cases in children and adults in Bangladesh (GBD04; GBD30). A comparison between ETEC and enteropathogenic *E. coli* (EPEC) diarrhea showed that both were significantly more frequent in African than in Asian sites (GBD14). Both ETEC and EPEC diarrhea episodes were associated with significant growth faltering in both regions. ETEC was found year-round in two geographically distinct regions in Bangladesh, where approximately 70% of the positive samples came from the surface water, planktons, and sediments with seasonal fluctuations. ETEC isolates showed high resistance to the macrolide antibiotics like erythromycin and azithromycin (ranges of 50–90%), as well as to ampicillin (85.7%) and ceftriaxone (71.4%) (GBD24). In a study to detect ETEC in Zambia, the novel Rapid LAMP-based Diagnostic Test (RLDT) was shown to be sufficiently sensitive (85–100%) and specific (98–100%) compared to quantitative PCR (qPCR) and was easy to implement (GBD19). The detection rate of ETEC (19%) was the same by both assays, with LT-ETEC (9%) being the highest.

There was a reportedly high resistance to the combination antibiotic sulfamethoxazole-trimethoprim of between 82 and 93% from ETEC, *Shigella,* and other *E.coli* isolates in a study in Zambia with about 3.5% of these isolates being multidrug resistant (to more than three antibiotics) (GBD07; GBD08). The prevalence of *Shigella* in Zambia by culture method was 13.6% (GBD10) and incidence of 30.6/1,000 child-years (GBD08), with serotype distribution across sites being 28–56% for *S. flexneri*, 22% for *S. sonnei*, 9% for *S. boydii,* and 7.5–12% for other *Shigella* spp. The incidence of ETEC was 30/1,000 child-years with children aged one to two years having the highest burden (GDB21).

A spatiotemporal cluster identification of areas at risk for specific diarrhea pathogens revealed that Lusaka, Zambia, had a significant Relative Risk of 3.7 of a child having diarrhea compared to other sites, with cases being associated with EPEC (36.8%), ETEC (20%), *Campylobacter coli* (22%), and *C. jejuni* (12%) infections (GBD22). An investigation into other co-infections (GBD29) revealed high prevalence of diarrheagenic *E. coli* (DEC) with EPEC (45%), EAEC ( 38%), and ETEC (28%) as the highest. Mono infections accounted for 38% of the total cases, while double infections accounted for 61% with 10% having more than four enteric pathogens. DEC exposure was detected in children younger than 12 months of age, indicating a need for early intervention to reduce post-diarrhea sequalae.

In Ghana, the prevalence of enteric pathogens among healthcare staff was evaluated in order to curb potential outbreaks originating from health centers. Of the healthcare workers who were screened, *Salmonella* spp. (6.6%) and *Klebsiella pneumoniae* (3%) were the most common enteric pathogens recovered, highlighting the need for routine surveillance and the importance of compliance with infection prevention and control measures (GBD18).

Samples from children younger than five years of age in India who were hospitalized at three sentinel surveillance hospitals in from April 2017 to December 2020 were tested by qPCR for 16 established etiological causes of diarrhea (GBD17). Results indicated that 48% of the diarrheal hospitalizations were among children aged 0–11 months with adenovirus as the leading cause of diarrhea (28.3%) followed by rotavirus (15.3%), *Shigella* (12%), and norovirus (11%). *Shigella* (22.7%) was the most common pathogen among children older than 23 months. Based on these findings, the rotavirus burden remains significant in India despite the national introduction of rotavirus vaccines.

In more regional studies in northern India, new qPCR-based detection assays for ETEC and *Campylobacter*, along with whole genome sequencing, were used to more accurately assess burden. Overall, ETEC was detected in 4.7% of all acute diarrhea cases tested with 44% being from children younger than 15 years of age (GBD15). Many of these isolates were resistant to the antibiotics ciprofloxacin and ceftriaxone. For *Campylobacter*, whole genome Ribosomal Multilocus Sequence Typing (rMLST) revealed 13 different sequence types that clustered into two clonal complexes for which data suggested a zoonotic source of human infections in this region of India (GBD20). In AMR studies, the continued emergence of multidrug resistant *S. typhi* strains in northern India remains a concern, highlighting the importance of improving typhoid vaccine coverage in India (GBD35).

Non-typhoidal *Salmonella* (NTS) in sub-Saharan Africa are a major cause of bloodstream infections among children and a growing public health concern, particularly in children younger than five years old. Children in Kenya (GBD27) presenting with fever for more than 24 h with or without diarrhea were enrolled in a study, and household contacts of index invasive NTS (iNTS) cases were also studied. Results indicate that the presence of NTS carriers within this study site represent a risk to younger age-groups, highlighting the need for vaccine use in the prevention of NTS cases among infants and children. Similarly, the SAiNTS study in Malawi (GBD32) is a prospective community cohort study designed to measure age-stratified acquisition of lipopolysaccharide O-antigen antibody and serum bactericidal activity to the main serovars causing iNTS (*S. Typhimurium* and *Enteritidis*) in children younger than five years of age. This study is ongoing and will ultimately provide valuable information on the epidemiology of enteric NTS and subsequent acquisition of immunity in children resulting from invasive and asymptomatic NTS disease.

Antibiotic prescribing practices for moderate-to-severe diarrhea (MSD) cases were assessed in the Gambia, Mali, and Kenya (GBD06). Results indicate that antibiotic prescriptions in these countries were associated with signs and symptoms inconsistent with World Health Organization (WHO) guidelines, suggesting the need for better antibiotic stewardship and clinician awareness on diarrhea case management in these settings. Consistent with the overuse of antibiotics in many parts of Africa, studies in Ethiopia (GBD34) and Zambia (GBD07; GBD08) presented concerning AMR data. These data indicate that animal and environmental isolates play an important role in the local dissemination of antimicrobial resistant *E. coli*. In Zambia, studies are underway to determine the prevalence of AMR strains among *Shigella* isolated from children under five years of age. This work complements previous studies documenting a significant level of AMR among ETEC strains recovered from infants and young children with MSD (GBD07).

## 3 Assessment of the value of vaccines against enteric pathogens

Assessing the value of vaccines and optimizing their impact can help drive investment and future demand, and several studies examined different aspects of potential new *Shigella* vaccines. For example, elevated levels of maternally transmitted antibodies to *Shigella* lipopolysaccharide (LPS) in 6-week-old Zambian infants waned significantly by 14 weeks (AVV11), indicating a potential vaccination window after 14 weeks. Interviews with health experts in Asia and Africa showed that an effective vaccine at 6 to 9 months of age was only considered a moderate priority (AVV01). Another driver of increased prioritization was potential impact on growth stunting. Given the strong correlation between adult height and wage income, the potential productivity benefits of a *Shigella* vaccine that reduces child growth faltering indicated that even a moderately effective vaccine could pay for itself due to productivity gains alone (AVV02).

Typhoid fever and diarrhea caused by ETEC are each responsible for a significant disease burden in children in LMICs and are further exacerbated by emerging AMR, and vaccines could play an important role in addressing these issues. An analysis of blood culture samples from children diagnosed with typhoid fever in Pakistan found that 46.1% of *Salmonella typhi* isolates were multidrug resistant; among children 5 to 14 years old, 44.2% were extensively drug resistant (AVV13). *S. typhi* and *S. paratyphi* resistance was also high in other age groups, and the proportion of resistant isolates rose between 2017 and 2019. When children in Pakistan aged 6 months to 10 years were immunized with a single dose of a typhoid conjugate vaccine it was found that 92.8% had seroconverted (defined as fourfold increase in anti-Vi-IgG) (AVV10). An ETEC vaccine could be beneficial in reducing increasing levels of AMR (AVV12), yet the most advanced vaccine candidate, in Phase 3 clinical trials, will not be available for at least five years. An ETEC vaccine could also help prevent travelers’ diarrhea (TD). To address difficulties in measuring ETEC vaccine impact on TD severity and incidence in clinical trials, a new disease scoring system has been proposed (AVV03) and may serve as a valuable new, more sensitive metric.

Recent approaches to the development of interventions against *Shigella* include a single dose of WRSs1, a live attenuated *S. sonnei* vaccine, which elicited robust antibody responses in serum and ALS (AVV06). These studies indicate multiple doses may be necessary to provide lasting immunity. A second vaccine approach utilizes defined antigens LTA1, the ETEC mucosal adjuvant double-mutant heat-labile toxin (dmLT), and an IpaD/IpaB fusion protein to generate the self-adjuvating vaccine L-DBF (AVV09). This protected mice from lethal challenge with multiple serotypes of *S. flexneri* and *S. sonnei*, indicating potential cross-protection. An alternative strategy is to use monoclonal antibodies (Mabs) for therapy rather than antibiotics. Peripheral blood mononuclear cells from participants in several clinical trials were screened for Mabs that recognize multiple *Shigella* serotypes to identify potentially cross-reactive antibodies (AVV07). These Mabs will be evaluated for the ability to inhibit adhesion and invasion of colonic epithelium.

Evaluation of cholera vaccines in endemic areas is complicated by outbreaks. Studies of volunteers after Shanchol^TM^ vaccination found the vaccine was immunogenic, with vibriocidal antibody titers declining quickly yet remaining elevated above baseline (AVV08). This suggests routine re-vaccination in high-risk areas may be a viable strategy since no empirical evidence supports an ideal time.

## 4 Preclinical evaluation of vaccine candidates and models of enteric diseases

*In vivo* and *in vitro* models to elucidate pathogenesis and develop interventions against enteric pathogens are being explored to facilitate vaccine development. The highly human-relevant enteroid mini gut model was used to investigate ETEC and *Shigella* pathogenesis. The role that co-expressed CFs play in ETEC was assessed. Adherence to human enteroids by clinical ETEC isolates expressing CFA/I and CS21 was reduced by deletion of CFA/I but not CS21 and was blocked by anti-CFA/I but not anti-CS21 (PRE01). Enteroids were used to quantify invasion of *S. dysenteriae*, *S. flexneri,* and *S. sonnei* using a gentamicin protection assay and intracellular bacteria were visualized by confocal microscopy (PRE08). The enteroid model was further used to identify the contribution of previously uncharacterized *S. flexneri* genes to gastrointestinal survival and virulence and to identify cellular changes that occur as a consequence of bacterial exposure to small intestine conditions (PRE02).

Antibodies from rabbits vaccinated with a quadrivalent *Shigella* bioconjugate had serum bactericidal activity (SBA) against the four component serotypes as well as cross-reactivity against *S. flexneri* 2b, 4a, and 4b (PRE011).

Residual reactogenicity has hampered advancement of some live attenuated *Shigella* vaccine candidates. Lipid A modifications introduced via ectopic expression of LpxE and/or PagL reduced the toxicity of *Shigella* LPS *in vitro* and *in vivo* (PRE05).

EtpA is a highly conserved adhesin found in diverse ETEC strains. Critical host cell binding epitopes in the C-terminal repeat region were identified by mass spectrometry and cryo-electron microscopy that inform vaccine development strategies (PRE17).

Success has been achieved in developing and refining a *Campylobacter* model in adult mice (>12 weeks of age) fed zinc-deficient diets and treated with antibiotics (PRE07; PRE21). This model exhibits the hallmarks of *C. jejuni* infection (colonization, weight loss, diarrhea associated with mucus and blood, and elevated mucosal inflammatory markers).

ETEC heat-labile (LT) and heat-stable (ST) enterotoxins were found to induce intestinal production of the epithelial alarmin IL-33 (an initiator of the inflammatory cascade) in a patent mouse assay (PRE09) and may present a novel therapeutic target and may help define correlates of ETEC morbidity and immune protection.

Preclinical evaluation of new enteric vaccine candidates is receiving much attention. The multiple epitope fusion antigen (MEFA), uses of a protein backbone fused with multiple other protective epitopes of the targeted pathogen. Given intramuscularly with the adjuvant dmLT, an ETEC MEFA vaccine, MecVax (PRE19), a *Shigella* MEFA (PRE03), and a cholera MEFA (PRE15) were each found broadly immunogenic inducing functional antibodies against vaccine antigens with reduction in intestinal colonization and protection against pathogen challenges.

The yield of *Shigella* IpaB for vaccine use has previously been unsatisfactory but may be improved using an XpressCF + TM cell-free protein synthesis platform with as much as 100-fold increase in yield of immunologically active IpaB (PRE20). Another current approach to broad coverage against *Shigella* is the use of candidates made up of synthetic oligosaccharides mimicking potentially protective determinants carried by the O-antigens of *S. flexneri* 2a and *S. sonnei* (PRE13). For ETEC, a combined formulation containing three representative Class 5 fimbrial adhesins elicited a functional immune response against all of the Class 5 ETEC strains tested (PRE12).

Efforts to identify proteins in GMMAs from non-typhoidal serovars *S. enteritidis* and *S typhimurium* revealed conserved proteins also present in *S. Typhi* with potential to elicit pan-*Salmonella* cross-reactive responses (PRE16). A live attenuated non-transmissible *S. typhimurium* vaccine was well tolerated and initiated robust immune responses against core O-polysaccharide and protected mice against a lethal challenge (PRE10).

## 5 Vaccine candidates in clinical trials and human challenge models

There are several enteric vaccine candidates in early- or mid-stage clinical evaluation or that are being studied in expanded immunological studies evaluating correlates of protection and duration of immunity. The safety, tolerability and immunogenicity ETVAX®, the most advanced ETEC vaccine, has been evaluated in several clinical trials in Africa. In one study, a high proportion of Zambian adults seroconverted to LTB and, in children (aged 6–9 months), the ¼ dose induced serologic responses to at least three vaccine antigens with no safety concerns (CLT08). This ¼ dose is currently being evaluated for efficacy in 6- to 18-month-old Gambian children, with the last vaccination dose administered in October 2022 (CLT01).

To assess the sustainability of immune responses, serum samples from a subset of participants were collected 200 to 400 days after vaccination with a recombinant CS6-based subunit (CssBA) prototype ETEC vaccine administered intramuscularly with dmLT (CLT03). Increased anti-LT IgG avidity was seen in participants who received 0.5 µg dmLT with no differences in avidity across CssBA doses. Anti-CS6 and anti-LT IgG remained significantly elevated in the long-term samples anti-CS6 compared to baseline samples suggesting ongoing antibody maturation.

Expanded analyses of a controlled human infection model (CHIM) were conducted to evaluate the efficacy of an *S. sonnei* GMMA-based vaccine (1790GAHB) (CLT04). Although the vaccine did not protect against shigellosis, results suggest circulating IgG levels or bactericidal activity are associated with protection against shigellosis, but the quality of 1790GAHB-induced antibodies may differ from those induced by *S. sonnei* infection. In related follow-on studies, an improved four-component formulation of the GMMA vaccine was developed and will be tested in European adults with a subsequent descending-age study planned in Africa (CLT10).

Another *Shigella* vaccine (SF2a-TT15) utilizing synthetic oligosaccharides (OS) covalently linked to a tetanus toxoid was safe and immunogenic in a Phase 1 study in Israeli adults (CLT05). A 10 µg OS dose induced long-lasting (2–3 years) functional humoral responses in the majority of subjects. The valency of a *Shigella* bioconjugate that previously demonstrated evidence of protection in a CHIM with the monovalent formulation target *S. flexneri* has been increased to include *S. sonnei*, S. *flexneri* 3a, and 6. This quadrivalent vaccine is being evaluated in a descending-age, dose-escalation study in Kenya (CLT02).

Updated analyses of an *S. sonnei*-rEPA conjugate vaccine in young adults and children identified that IgG anti-*S. sonnei* LPS of at least 4.5 ELISA units corresponded to 52% protection in Israeli children aged 2 to 4 years (CLT12). This association was retained after adjusting for age and population group differences among Israeli children and adolescents. A related analysis of serum antibody results from prior efficacy trials of the *S. sonnei*-rEPA vaccine in Israel also pointed to serum IgG anti-*S. sonnei* LPS threshold antibody levels as a predictor of vaccine field efficacy (CLT14).

A live attenuated, oral vaccine, designated ShigETEC, has been manufactured to produce protection against ETEC and *Shigella* via a toxoid fusion protein to induce protective antibody responses in a serotype-independent manner due to the lack of O-antigen expression, enabling a response to minor shared and conserved antigens, respectively (CLT13). ShigETEC was evaluated in Hungarian adults and was well-tolerated up to a 5x10^10^ CFU/dose. CHIM studies with *Shigella* and ETEC strains are planned.

The Vi-tetanus toxoid conjugate vaccine (Vi-TT) against typhoid fever was evaluated in a cluster-randomized, double-blind trial in Bangladeshi children aged 9 months to 16 years (CLT07). Overall efficacy was 56% after 24 months with an additional 24 months of follow-up planned. These data will help guide efforts to determine the timing of a booster dose. A typhoid conjugate vaccine manufactured by EuBiologics (EuTCV) is currently being evaluated in single- and multi-dose presentations in a Phase 3 study (CLT15).

Researchers evaluated the motivating factors and experiences of CHIM participants and found the primary factors for participation were money, positive staff interactions, and food (CLT11). Additionally, most volunteers enjoy participating, discovering new things about their health status and did not feel that their participation negatively affected their well-being. Consideration of these factors is important to ensure a continued ability to successfully recruit CHIM participants.

## 6 Host parameters and genomics that predict responses to infection and disease

The LT enterotoxin plays an important role in intestinal inflammation. ETEC infections have been linked to stunting and malnutrition, conditions that may be driven by inflammation. Challenge of human volunteers with an LT + ETEC strain was followed by significant increases in inflammatory mediators demonstrating that infection with LT-producing strains leads to significant inflammation independent of acute presentation (GEN01). This observation is consistent with field data suggesting that even asymptomatic infection with ETEC strains producing LT toxin can cause significant intestinal inflammation and contribute to EED and stunting. In additional laboratory-based studies, LT was found to modulate many genes required for biogenesis of the brush border, the major absorptive surface of the small intestine. Infant mice challenged with toxigenic wild-type ETEC (but not nontoxigenic mutants) developed enteropathic changes in the surface of the small intestine, while maternal vaccination with LT generated antibodies that protected infant mice from enteropathy. This suggests that an effective ETEC vaccine could prevent both acute illness as well as long-term morbidity (GEN02).

Biomarkers are important for assessing enteric infection severity and impact. In continuing efforts to identify potential biomarkers of disease severity, recent ETEC CHIMs data indicate that plasma levels of proguanylin decreased significantly from baseline in volunteers experiencing severe diarrhea, potentially offering a biomarker for disease severity following infection with ST-producing ETEC (GEN06). In related field studies examining biomarkers of cholera severity, blood group O has been associated with higher risk of severe cholera disease. Individuals from a cholera-endemic area in Zambia were vaccinated with two doses of Shanchol™ and tested to determine their HBGA and secreta status in saliva. No significant differences in seroconversion status were observed among secreters compared to non-secreters or among those with blood group O verses non-O (GEN03), suggesting ABO blood group may not modulate immune responses to oral cholera vaccine. In novel psychomotor assessments, sleep and acute infectious diarrhea were examined for their impact on vigilance performance in a CHIM with ETEC. Interestingly, Illness severity, independent of sleep loss, negatively impacted performance which may have important implications for individuals infected with ETEC in military operational settings, as well as in civilian workplace and educational environments (GEN09).

Improved understanding of immune responses to ETEC infection and immunization is important for optimal vaccine development. ETEC infection or vaccination induces T follicular helper (Tfh) cell responses in peripheral blood manifesting primarily as a Th17 phenotype, consistent with strong IgA antibody promoting capacity. The possibility to monitor Tfh responses in peripheral blood provides new possibilities to study germinal center reactions in diarrheal disease patients that may enable a better assessment of immune markers for protective ETEC immunity (GEN05). Similarly, new studies in five children’s hospitals in Zambia will help improve understanding of immune responses to key vaccine target antigens in children naturally infected with *Shigella*. This work should provide helpful insights into the burden of shigellosis in Zambia, as well as serum markers of immune protection and/or reduced risk of *Shigella*-associated illness to help drive vaccine development (GEN08).

Lymphostatin, a virulence factor of EPEC and non-0157 serogroup EHEC, inhibits anti-CD3 and anti-CD28 activated proliferation of CD4 + and CD8 + T cells and blocks synthesis of IL-2, IL-4, IL-5, and IFN-γ without affecting cell viability. This inhibition was not observed in T cells activated by phorbol 12-myristate 13-acetate and ionomycin, indicating that lymphostatin targets T cell receptor signaling. From analysis of CD69 expression, lymphostatin seems to suppress T-cell expression. This suppression appears to be related to a general blunting of cellular phosphorylated kinases (GEN04). These data provide insights into the mode of action of this novel bacterial virulence factor from EPEC and EHEC on host immunity.

The immunological basis for the broad protection provided in Finnish travelers to Benin by the oral ETEC vaccine candidate ETVAX needs to be better understood. To investigate this observation further, an ETEC proteomic array, developed by Antigen Discovery Inc., was used to show that volunteers without severe TD had broader IgA/IgG responses to vaccine antigens in general and more specifically to class 5 fimbriae not present in ETVAX but sharing antigen epitopes with the CFA/I vaccine component. These results suggest that broader class 5 fimbrial antibody responses may be associated with a decreased risk of ETEC diarrhea (GEN07).

## 7 Application of new omics technologies for characterization of emerging pathogens and host responses

Several studies have been conducted using new genomic and proteomic approaches for characterizing host responses to bacterial enteric pathogens and identifying correlates of protection and novel candidate antigens. One study employed a novel mutiplexed assay to compare *Shigella* antibodies in children from endemic regions with subjects from a CHIM study (NOT02). A similar study used a systems serology approach to define humoral correlates of protection from CHIM study subjects (NOT03). Transcriptional profiling of various ETEC strains revealed expression patterns associated with particular metabolic states as well as candidate cell surface proteins with potential to stimulate broad protection (NOT06). Finally, the well-established TaqMan Array Card platform was used to analyze samples from an ETVAX TD trial and serve as a more sensitive method than traditional culture to identify ETEC from clinical samples (NOT13).

High-throughput analysis of a large amount of sequence data estimated the extent that CFs are shared across other *E. coli* and related species (NOT08). Hidden Markov Models have been used to determine the spread of ETEC CFs. Phylogenetic analyses were employed to describe the prevalence, distribution, and relatedness of CFs across bacterial species. Mutli-locus variable repeat analysis patterns from recent toxigenic *Vibrio cholerae* O1 Nigerian patient isolates demonstrated a *founder flush* pattern with the founder genotype giving rise to multiple other genotypes as the outbreak expands and spreads over time and space (NOT09). Large-scale BLAST score ratio and phylogenomics analysis demonstrated that CCH060 (*Sf6*) was the most genomically distant and exhibited the greatest amount of unique genomic features among three archetype *Shigella* isolates, 2457 T (*Sf*2a), J17B (*Sf*3a), and CCH060 (*Sf*6) (NOT10). Pathogenomic analysis of *Shigella* isolates from South Asia and sub-Saharan Africa revealed *S. sonnei* and protein antigen candidates (relative to other species and serotype-specific targets) to be conserved vaccine targets and identified convergent evolution of resistance against ciprofloxacin (NOT11). Moreover, serotype switching in *S. flexneri* was observed, which may lead to immune escape from O-antigen based vaccines.

Proteome microarray technology can be used to characterize the host antibody response to diarrheal pathogens. In a Zambian pediatric trial of ETVAX, pan-diarrheagenic *E. coli* proteome microarrays were used to elucidate cross-reactive IgG responses against non-vaccine class 5 fimbriae proteins that may provide protection from ETEC and other pathotypes (NOT01). Two *Campylobacter jejuni* studies utilized protein microarrays. One examined IgY responses in immunized breeder chickens and their broiler offspring (NOT05), and another analyzed IgA and IgG responses in a *C. jejuni* CHIM (NOT07). Both studies identified significant antibody reactivity against *Campylobacter* outer membrane and flagellar proteins, the former highlighting the benefit of passive antibody transfer and the latter the variation in human responses to different strains. The mucosal antibody response in understudied biological specimens such as colostrum and breast milk was also explored utilizing a multipathogen protein microarray to characterize the breadth and magnitude of secretory IgA and IgG responses in human milk (NOT04). Antibodies were associated with reduced risk of infection from rotavirus A, *Shigella,* and adenovirus 40/41 in the breastfeeding infants of mothers from Bangladesh. These early studies lay the groundwork for future vaccine antigen discovery.

## 8 Novel adjuvants, vaccine delivery platforms, and immunization strategies

A study was conducted on using pre-exposure prophylaxis as a way to provide protection from ETEC (ADJ04). Heavy chain nanobodies which targeted ETEC adhesins or CFs were fused with IgG to generate nanobody molecules which demonstrated cross-protective potency against eleven major pathogenic ETEC strains *in vitro* and demonstrated a significant reduction in bacterial colonization in animals.

Photochemical inactivation is being studied as a novel platform for killed whole cell vaccines (ADJ05). Unlike formalin inactivation, which is known to induce chemical modifications of proteins which can affect antigenicity, photochemical inactivation was shown to reproducibly yield inactivated bacteria which were able to generate equivalent or greater IgG levels compared to formalin-killed bacteria in mice as well as potentially expanding the antigenic repertoire presented to the immune system.

Using a Multiple Antigen Presenting System (MAPS), four *Shigella* MAPS vaccines were developed, for *S. flexneri 2a, 3a, 6*, and *S. sonnei,* using a *Shigella* surface protein as a carrier in combination with OSPs (ADJ08). *In vitro* evaluation of the quadrivalent *Shigella* MAPS vaccines demonstrated equivalent immunogenicity to each of the monovalent vaccines, generating high titers of functional antibodies against each type O antigen.

Another study examined the effect of HIV status, viral load, and CD4 count had on the immune response to oral cholera vaccine (Shanchol™) (ADJ01). Participants who were HIV-positive, had high viral load, and low CD4 counts showed reduced immunogenicity to Shanchol™, potentially affecting the protective efficacy of the vaccine. Further studies are needed to inform appropriate policy and practice for cholera vaccination.

In a study on the role that immunization route played in T-cell responses to an ETEC vaccine candidate, mice were immunized with dmLT and CssBA by intramuscular or sublingual delivery (ADJ07). Sublingual immunization was the primary source of IL-17 T-cell responses, confirming that this cytokine response was route-specific, while IL-2-secreting T cells were observed after both routes of immunization.

A reduced protein concentration formulation of the dmLT adjuvant was evaluated in order to reduce waste and the complexity of mixing with vaccines for clinical study (ADJ03). The dmLT concentration was reduced 50-fold from 1 mg/mL to 20 μg/mL, and 0.05% polysorbate 80 (PS80) was added while keeping the other excipients constant. The freeze-dried formulation was shown to have improved stability under accelerated heat stress at 40 °C for four weeks.

The comparability of *in vitro* and *in vivo* evaluations of dmLT were explored (ADJ06). Stable dmLT formulations were subjected to stress and then were evaluated in animals. Both stable and stressed dmLT samples were co-formulated with ETEC or inactivated poliovirus vaccines and administered to mice via intradermal or sublingual immunization. Vaccine efficacy was affected more by the immunization route and vaccine antigen than the predicted *in vitro* stability of dmLT. While biochemical methods are necessary for product characterization, they are not necessarily predictive of vaccine outcome. Both biochemical and animal model testing are necessary when optimizing the vaccine formulation.

## 9 Strategies for combination/co-administered vaccines

The future commercial success and uptake of new enteric vaccines may be improved by combining or co-administering vaccine antigens to protect against multiple pathogens at the same time. There are no licensed vaccines available for use against either NTS and *Campylobacter* species. A trivalent outer membrane vesicle (OMVs)-based immunogen was immunogenic and protective against both NTS and *Campylobacter* in a mouse model (CMB01). GMMA are outer membrane exosomes from Gram-negative bacteria that have been used as a “plug and play” technology for the development of effective multicomponent vaccines (CMB02). A combination of iNTS-GMMA and Vi-CRM197 that could result in an effective and affordable vaccine, iNTS-TCV, may be a viable option for a sustainable iNTS vaccine in sub-Saharan Africa (CMB03). Optimal batches of the iNTS conjugate and the increase in antibody titer induced was found to be dependent on the conjugation method used. This trivalent iNTS/typhoid vaccine will be tested for toxicology in other animal models (PRE06).

## 10 Conclusion

The plenary content and poster presentations from the 2022 VASE Conference summarized here provides a snapshot of the extensive research currently taking place around the globe in the enteric vaccine field. Topics ranged from the burden of disease and assessing the value of vaccines to preclinical and clinical evaluations of vaccine candidates, and included research on predicting responses to infection and disease and characterizing host responses, novel adjuvants, and strategies for combination vaccines.

Several themes emerged through the conference presentations, highlighting areas to prioritize for future research in the field. It is clear that enteric diseases remain a substantial public health burden in low-resource settings, and new vaccines, diagnostics, and other interventions are urgently needed. Innovative approaches to improving vaccine efficacy and delivery are likely to increase uptake of new enteric vaccines when they become available. In addition, vaccines against a broader scope of pathogens, potentially given as combinations, will help address diarrhea and AMR more effectively. These presentations and subsequent discussions at the 2022 VASE Conference should help to accelerate the development and future introduction of vaccines against *Shigella*, ETEC, *Campylobacter*, non-typhoidal *Salmonella*, and other neglected enteric pathogens.

## Funding

This work was supported by the Bill & Melinda Gates Foundation [INV-000875]. Under the grant conditions of the Foundation, a Creative Commons Attribution 4.0 Generic License has already been assigned to the Author Accepted Manuscript version that might arise from this submission.

## Copyright Statement

Some of the authors are U.S. Government employees. This work was prepared as part of their official duties. Title 17 U.S.C. §105 provides that ‘‘Copyright protection under this title is not available for any work of the United States Government.” Title 17 U.S.C. §101 defines a U.S. Government work as a work prepared by a military service member or employee of the U.S. Government as part of that person’s official duties.

## Disclaimer

The opinions expressed in this article are those of the authors and do not reflect the view of the Department of the Navy, Department of Defense or the United States Government. There are no restrictions on its use.

## Declaration of Competing Interest

The authors declare that they have no known competing financial interests or personal relationships that could have appeared to influence the work reported in this paper.

## Data Availability

This is a conference report and does not contain original data.

